# Psychometric Properties and Convergent Validity of the Shirom–Melamed Burnout Measure in Two German-Speaking Samples of Adult Workers and Police Officers

**DOI:** 10.3389/fpsyt.2019.00536

**Published:** 2019-08-02

**Authors:** René Schilling, Flora Colledge, Serge Brand, Sebastian Ludyga, Markus Gerber

**Affiliations:** ^1^Department of Sport, Exercise and Health at the University of Basel, Basel, Switzerland; ^2^Center for Affective, Stress and Sleep Disorders, Psychiatric Clinics of the University of Basel, Basel, Switzerland; ^3^Department of Psychiatry, Kermanshah University of Medical Sciences (KUMS), Substance Abuse Prevention Research Center and Sleep Disorders Research Center Kermanshah, Kermanshah, Iran

**Keywords:** burnout, validation, psychometric properties, internal consistency, mental health, stress

## Abstract

Burnout is considered an occupation-related psychological syndrome consisting of emotional, physical, and cognitive exhaustion. To assess dimensions of burnout, the Shirom–Melamed Burnout Measure (SMB*M*) is widely used, but its validity and reliability have rarely been examined in adult samples. The aim of this study is to examine the psychometric properties of the German version of the SMB*M* in two independent samples of adults. In total, 311 adult workers and 201 police officers completed the SMB*M*, and questionnaires related to perceived stress and mental well-being. Descriptive statistics, internal consistency, convergent validity, and factorial validity were assessed for both samples, separately for male and female participants. The German SMB*M* had adequate psychometric properties and sufficient convergent validity. In confirmatory factor analyses, we found a good fit for both the first- and second-order model. Furthermore, measurement invariance across gender was observed in both samples. Although the SMB*M* is a popular instrument among burnout researchers, this study demonstrates for the first time that the SMB*M* can be considered a valid and reliable tool to assess burnout symptoms in both male and female adults and across different professional groups. Furthermore, with its 14 items, the SMB*M* is a succinct and economic self-assessment tool for symptoms of burnout.

## Introduction

Burnout can be defined as an occupational syndrome consisting of emotional, physical, and cognitive exhaustion (1). While there is broad consensus that people with burnout require medical and psychiatric treatment, there has been constant debate as to whether burnout should be considered a specific and well-defined psychiatric disorder, an epiphenomenon of a major depressive disorder (ICD-10: F33.xx), an adjustment disorder (ICD-10: F43.xx) (2–7), or a form of chronic fatigue syndrome (ICD-10: G93.3). Bianchi et al. (8) argue that there is such a strong overlap between burnout and depression that burnout should not be considered as a specific job-related phenomenon, but rather as a depressive condition. However, others argue that the two constructs are distinct (9) and that burnout syndrome should be given the status of an occupational disease (10, 11). Currently, there are no conclusive diagnostic criteria (11, 12), and to date, the condition is not included in the Diagnostic and Statistical Manual of Mental Disorders (5th Edition) (DSM 5) (13). In the 11th version of the International Classification of Diseases (ICD-11), however, “burnout” is classified under QD85 and is defined as “a syndrome conceptualized as resulting from chronic workplace stress that has not been successfully managed” (see https://icd.who.int/browse11/l-m/en#/http://id.who.int/icd/entity/129180281). The ICD-11 definition highlights that burnout is a work-related phenomenon and thus not suitable for the description of experiences in other life domains.

The Swedish Health System recognizes burnout as a psychiatric disorder; therapeutic interventions and sick leave for affected individuals are standard treatment forms (14, 15). Moreover, a recent Europe-wide study concludes that 9 of 23 European countries currently consider acknowledging burnout as an occupational disease (16) [see also Refs. (10, 11, 17)]. Irrespective of diagnostic issues, we observe that burnout is a serious public health problem and therefore a cause for concern for policy makers, patients, and health insurance organizations (18).

To assess dimensions of burnout, the Maslach Burnout Inventory (MBI) is the most widely used instrument (19, 20). Maslach et al. (21) defined burnout as a (multidimensional) psychological syndrome consisting of emotional exhaustion, depersonalization/cynicism, and reduced personal accomplishment. Consequently, burnout is often considered synonymous with the definition provided by Maslach and colleagues (21–23). This also holds true for the new ICD-11 definition, where burnout is characterized by three dimensions, namely, “the feelings of energy depletion or exhaustion, increased mental distance from one’s job, or feelings of negativism or cynicism related to one’s job, and reduced professional efficacy.” Nevertheless, the theoretical and scientific basis of the MBI has been questioned (19, 20, 24), especially in light of the fact that the three burnout dimensions were not deducted theoretically but are the result of exploratory factor analysis. In addition, it has been argued that the depersonalization/cynicism and reduced personal accomplishment subscales do not adequately represent the core of the burnout construct.

By contrast, Shirom, Melamed, and colleagues took the basic tenets of the Conservation of Resources (COR) theory (25, 26) into consideration. The resulting definition of burnout included an individual’s feeling of being emotionally exhausted, physically fatigued, and cognitively worn-out (18, 27). Briefly, the COR theory assumes that people have a basic motivation to obtain, retain, and protect the resources that they value (28, 29). Accordingly, the chronic depletion of an individual’s energetic resources following prolonged exposure to emotionally charged demands has been identified as the unique content of the burnout construct (30–32). More specifically, physical fatigue refers to an individual’s feelings of tiredness and low levels of energy in carrying out daily tasks at work (or in general life) (p. 330) (27). Emotional exhaustion, on the other hand, describes the interpersonal aspect of burnout, “namely, feeling that one lacks the energy needed to invest in relationships with other people at work” (p. 330) (27). Finally, cognitive weariness describes the phenomenon of slower thinking and impaired mental agility. Melamed et al. (27, 31, 33) further hypothesized that this definition of burnout is distinct from a temporary state of fatigue, which generally disappears after a reasonable period of rest. Furthermore, Lundgren-Nilsson et al. (20) claimed that “this conceptualization of burnout has been proven useful, not only to measure burnout in working populations, but also in clinical populations of patients seeking medical care due to stress-related exhaustion” (p. 1).

Using Shirom and Melamed’s (18, 27) definition of burnout, research has shown associations between burnout and both physiological and psychological health outcomes. Physiologically, higher burnout scores are associated with increased cardiovascular risk factors, including increased fasting glucose and cholesterol levels (31, 34–36), increased cortisol levels throughout the day (33), an elevated cortisol awakening response (37), increased leukocyte adhesiveness (32), increased inflammatory markers (35, 38), increased risk of developing type 2 diabetes (39, 40), increased risk of musculoskeletal pain (41), and a higher likelihood of infertility (42). As regards psychological dimensions, data from vocational students and adult workers have shown that higher burnout levels are associated with reduced life satisfaction and quality of sleep (33, 43, 44). Similarly, significant associations have been found between burnout and depression, although the level of overlap varied considerably (38, 45–48). Moreover, in the clinical setting, Glise et al. (14) showed that among individuals with diagnosed job-related exhaustion disorder, ∼90% displayed severe burnout scores. Finally, a multimodal treatment approach has been shown to lead to a reduction of burnout symptoms in the majority of patients (15, 49).

The Shirom–Melamed Burnout *Questionnaire* (SMB*Q*) was devised to assess this COR-inspired definition of burnout. The questionnaire consists of eight items to assess symptoms of physical fatigue and emotional exhaustion (e.g., “I feel physically exhausted.”) and four items to assess tension (e.g., “I am tense.”) and listlessness (e.g., “I feel sleepy.”), respectively (30, 31). A distinction between tension and listlessness was made because the development of burnout was originally considered as a two-phase process, with tension being predominant in the early stages when active and direct coping strategies are employed to enhance and protect resources, and listlessness being characteristic of the more advanced stages when indirect and inactive coping prevails and burnout becomes more closely linked with apathy and depression (31). Answer options on the SMB*Q* 7-point Likert scale range from 1 (almost never) to 7 (almost always), with higher scores reflecting a higher degree of self-rated burnout. Norlund et al. (50) employed the SMB*Q* among a sample of 1,000 participants representative of the general population in Northern Sweden: Using an (arbitrary) cut-off of ≥4.0, the authors showed that 9.9% of all men and 15.9% of all women reported high burnout levels, while the level of burnout decreased with age across both genders. Furthermore, in another epidemiological study with 2,694 health care and social insurance workers from the Gothenburg region (51), the prevalence of employees reporting high burnout (≥4.0) was considerably higher (24%), indicating that burnout prevalence rates might vary strongly as a function of age, gender, job, and sample.

To cope with some methodological issues, the Shirom–Melamed Burnout *Questionnaire* (SMB*Q*) was revised to form the 14-item Shirom–Melamed Burnout *Measure* (SMB*M*). In contrast to the SMB*Q*, the SBM*M* is composed of three subscales, namely, emotional exhaustion, physical fatigue, and cognitive weariness (32, 33). While the items of the first two dimensions were identical to those of the SMB*Q*, items related to cognitive weariness were added to assess the cognitive component of burnout (e.g., “My head is not clear.” or “It is hard for me to think about complicated things.”). This extension seemed plausible as cognitive weariness has been defined as a core dimension of burnout (18, 27).

Both the SMB*Q* and SMB*M* are employed in burnout research (see literature presented above), and the SMB*M* has been translated from English into several languages, including Czech, French, German, Hebrew, Polish, Russian, and Spanish (see www.shirom.org/arie/index.html#). However, the validity and psychometric properties of these translations have not been evaluated systematically. What is known so far is that the SMB*Q*/SMB*M* overall indices and subscales have satisfactory internal consistency (14, 19, 24, 30–31, 32, 37, 40, 52), with Cronbach’s alpha values generally exceeding accepted standards (α ≥ 0.70) (53). Furthermore, the SMB*Q*/SMB*M* have a relatively high time stability, with correlations of *r* ≥ 0.50 across follow-up periods of several years (39, 40, 54). With regard to the convergent validity, evidence suggests that burnout is closely associated with individuals’ chronic stress exposure (19, 50, 52, 55–57). Moreover, the SMB*M* and other burnout measures such as the MBI were reasonably correlated with each other. As expected, particularly high correlations were observed between the SMB*Q*/SMB*M* and the emotional exhaustion subscale of the MBI (19, 35). As reported previously, several longitudinal studies have confirmed that the SMB*Q*/SMB*M* instruments were able to predict specific physiological variables in the expected directions (e.g., cardiovascular disease risk factors, inflammatory biomarkers), thus underscoring the predictive validity of these questionnaires (27). Evidence has also suggested that at least moderate correlations exist between the SMB*Q*/SMB*M* and symptoms of depression and anxiety (45, 52). Preliminary results support the factorial validity of the SMB*M*: In a study with 717 Chinese hospital nurses, Qiao and Schaufeli (23) showed that a three-factor model achieved sufficient model fit, with the three factors being strongly correlated (*r* = 0.61–0. 73, *p* < 0.001). Moreover, in a sample of 214 Canadian employees, Sassi and Neveu (24) observed that a three-factor model resulted in a better model-fit than a one-factor solution. Again, a good fit was found between the three-factor model and the empirical data. Moreover, the three factors were moderately-to-strongly associated with each other (*r* = 0.38–0.58, *p* < 0.001). Finally, Lundgren-Nilsson et al. (20) used both Confirmatory Factor Analysis (CFA) and Rasch analysis to assess the construct validity of the SMB*Q*; their analyses showed that after removal of the items representing the tension subscale, an 18-item version of the SMB*Q* satisfied modern measurement standards. Most importantly, a cut-off of ≥4.40 for severe or clinically relevant burnout was suggested. With this cut-off, 83.4% of their clinical sample of patients suffering from job-related exhaustion disorder were placed above the cut, whereas 86.5% of the general population sample of health care and social insurance workers were categorized below the cut.

In summary, preliminary evidence supports the validity of the SMB*M*, while studies examining the psychometric properties and validity of the different language versions of the SMB*M* are still rare. To the best of our knowledge, only the French and Chinese versions of the SMB*M* have been examined systematically.

Given this background, the main purpose of the present study was to validate the German version of the SMB*M* across two different samples. We hold that the present study is important for several reasons: First, many scholars have used the SMB*M* during the last 25 years to assess burnout symptoms, and this holds true in German-speaking samples (44, 58, 59). Second, although it is well documented that men and women differ with regard to burnout prevalence (44, 50, 57), we are not aware of any study examining whether the psychometric properties of the SMB*M* apply equally across genders among adult workers.

Four hypotheses were formulated: First, we expected that women would show higher burnout scores than men (44, 50, 57). Second, we expected that adequate internal consistency would be found for the SMB*M* in both populations and both male and female. More specifically, we expected that all inter-item correlations would be ≥0.20. We also expected that Cronbach’s alpha values would be ≥0.70. Finally, we expected that item-total correlations would be ≥0.30 (23, 24, 32). Third, we expected to find adequate convergent validity in male and female participants and across both study populations. That is, we hypothesized that the SMB*M* subscales and the SMB*M* overall index would be moderately to strongly correlated with perceived stress (positive correlation) (44, 45, 48). Fourth, with regard to factorial validity, we expected that a three-factor model would produce adequate model fit (23, 24) and that both a first- and second-order model would fit well with the empirical data (24). In line with previous research (23, 24), we expected good factor loadings (≥0.55) across all items on the corresponding factors [see Ref. (60)] and at least weak measurement invariance across genders (more information regarding types of measurement invariance is provided in the *Materials and Methods* section).

## Materials and Methods

### Sample 1: Adult Workers

#### Participants and Procedures

The first study population was composed of adult workers who were recruited *via* exercise and health science students (*N* = 87) of the University of Basel, who took part in an introductory course in research methodology. Every student was asked to provide the names and email addresses of 6–12 people (no relatives) who would be willing to take part in an online survey. In order to obtain a broad sample, each student was asked to provide the names of a total of 12 persons from a variety of professional groups: a) with vocational education and training working in the primary (farming, forestry, hunting, mining, fishing) or secondary sectors (industry, construction industry), b) without higher education working in the tertiary sector (trade, transport, warehousing, hospitality, gastronomy, services), and c) with higher education working in the tertiary sector. For each of the three categories, students had to list one male and female person, and one person younger and one person older than 50 years. In total, the students suggested 756 potential participants (407 men, 349 women; on average 8.7 suggestions per student). Written informed consent was obtained from all participants, and the local ethics committee approved the study (EKNZ: 240/12). After two reminders, 311 adult workers completed the online survey (41.1% response rate).

#### Burnout

To measure symptoms of burnout, the participants answered the SMB*M* (32), which consists of 14 items that have been described in detail in the introduction section. The German version was downloaded from the homepage of Arie Shirom (www.shirom.org/arie/index.html; see **Supplementary online material**).

#### Perceived Stress

We employed the 10-item Perceived Stress Scale (PSS) (61) to measure participants’ levels of perceived stress. The PSS consists of 10 items and assesses stress during the past month. Participants report the frequency with which they find their lives unpredictable, uncontrollable, and overwhelming (e.g., “During the last month, how often have you been upset because of something that happened unexpectedly?,” “During the last month, how often have you felt that things were going your way?”). Answering options ranged from 1 (never) to 5 (very often). Higher scores are indicative of more pronounced subjective stress perceptions. The PSS proved to be a reliable und valid instrument in previous research (62, 63). In our population, we found a Cronbach’s alpha of α = 0.75.

#### Occupational Stress

We used the 11-item Job Content Questionnaire (JCQ) to assess an imbalance between demands and control at work (64). To assess job-related demands, participants answered five items on a 4-point Likert scale ranging from 1 (never) to 4 (often). For instance, we asked participants whether their job requirements include very fast or hard work or whether they have to accomplish large amounts of work. A sample item is: “My job requires me to work very hard.” In addition, participants completed six items to assess their perceived level of control at work. A sample item is: “I have freedom to make decisions about my job.” For each domain, we calculated a subscale score by summing up the values of each item, with higher scores being indicative of higher demands or control at work. We used the following formula to obtain the JDC ratio: job demand/(job control × 0.8333). In addition, we used the 16 items from the Effort-Reward Imbalance (ERI) questionnaire to assess job-related effort and reward (65). We assessed effort at work with five items and reward with 11 items, all of which were anchored on a 5-point Likert-scale. Items were completed in a two-step process. Participants first indicated whether they agreed or disagreed with the item content, describing a typical experience of their work situation. Items were scored 1 if participants did not experience a specific type of situation. If they did experience this type of situation, participants indicated how stressful each experience usually is for them, with response options ranging from 2 (not distressing) to 5 (very distressing). Sample items for the effort scale are: “I have a lot of responsibility in my job” or “I have many interruptions and disturbances in my job.” Sample items for the reward scale are: “I receive the respect I deserve from my superior or a similarly relevant person.” or “Considering all my efforts and achievements, my job promotion prospects are adequate.” Items were summed to obtain subscale scores for the effort and reward domains, with higher scores reflecting higher effort or reward. Because of the unequal number of items, we used the following formula to generate the ERI ratio: effort/(reward × 0.4545). Evidence for the validity and reliability of this instrument has been presented previously (65).

#### Depressive Symptoms

We applied the Depression subscale of the Hospital Anxiety and Depression Scale (HADS) to measure self-perceived depressive symptoms (66). The depression subscale of the HADS consists of seven items, asking participants about mood changes that may occur during the course of depression (e.g., “I still enjoy the things I used to enjoy.”). This instrument was originally designed for nonpsychiatric populations. Answers were given on a Likert-scale with four response options, from 0 (never) to 4 (almost always). Previous investigations have shown that the HADS has good psychometric properties and can be considered a valid tool to assess depressive symptoms. Items were summed to obtain an overall index, with higher scores being indicative of higher depressive symptoms. The Cronbach’s alpha was α = 0.71 in our population.

#### Statistical Analyses

Univariate analyses of variance (ANOVA) were used to examine gender differences. Correlational analyses were used to examine homogeneity and total correlations of all items. Internal consistency was measured with Cronbach’s alpha coefficient. Correlations were employed to test convergent validity. Finally, factorial validity was tested by means of CFA. Our expectation was that the 14 items would load on three different factors (six items on physical exhaustion, five items on cognitive weariness, and three items on emotional exhaustion). Accordingly, our three-factor model contained at total of 14 observed variables that were linked to three latent constructs. Maximum likelihood (ML) was applied to estimate the parameters. Moreover, we inspected multiple fit indexes to judge the fit between the empirical data and the theoretical model data (67). Simultaneous multiple group comparisons were used to test invariance of the measurement model across gender. As recommended by Byrne (68), good model fit is achieved if the normed fit index (NFI) is ≥0.95, the comparative fit index (CFI) is ≥0.95, the Tucker Lewis Index (TLI) is ≥0.95, and the root mean square error of approximation (RMSEA) is ≤0.05. As recommended by Comrey and Lee (60), standardized factor loadings should be interpreted as follows: ≥0.71 = excellent, ≥0.63 = very good, ≥0.55 = good, ≥0.45 = fair, and >0.32 = poor. CFA are performed with AMOS^®^ 24 (IBM Corporation, Armonk NY, USA), all other analyses with SPSS^®^ 22 (IBM Corporation, Armonk NY, USA). We compared the default model against a model which assumed configural (same pattern of fixed and free factor loadings across gender), weak (invariant factor loadings across gender), strong (invariant factor loadings and intercepts across gender), and strict (invariant factor loadings, intercepts, and unique factor variances across gender) measurement invariance in order to test measurement invariance across gender (69). We used Δχ^2^ to examine the fit of different models, with nonsignificant Δχ^2^-test scores indicating that the more restricted model fitted better with the empirical data.

## Results

Sample 1 was composed of 161 male and 150 female participants. The mean age was *M* = 42.64 years (*SD* = 14.02; range, 19–67 years). Participants reported a mean job experience of *M* = 21.61 years (*SD* = 13.95; range, 1–47 years). All participants were employed for at least 50% (*M* = 88.01%; *SD* = 17.87; range, 50–100%), with 60.1% in full time employment. The sample had a mean body mass index (BMI) (height in cm/body weight in kg^2^) of *M* = 23.87 (*SD* = 3.6), with 33.40% of the sample (*n* = 104) being classified as overweight (BMI ≥ 25). Moreover, 40.5% (*n* = 126) reported that they have children living at home, 1.9% (*n* = 6) had responsibility as a caregiver for a person in need of care, and 8.4% (*n* = 26) reported shift work. With regard to participants’ highest level of education, one person (0.3%) finished compulsory school without additional training, 42.5% (*n* = 132) completed vocational education and training, 9.6% (*n* = 30) completed academic high school, and 47.6% (*n* = 148) completed higher education. Finally, 14.5% (*n* = 45) reported that they are smokers, whereas 1.9% (*n* = 6) reported taking antidepressant medication.

In sample 1, we found a mean score of the SMB*M* overall index of 2.42 (*SD* = 1.00) (**Table 1**). In total, 5.8% (*n* = 18) of the participants had a burnout score that can be deemed clinically relevant (≥4.40). We did not find significant gender differences with respect to any of the SMB*M* overall index and subscales (**Table 1**). A χ^2^-test showed that a similar portion of women (*n* = 9, 6.0%) and men (*n* = 9, 5.6%) reported clinically relevant burnout symptoms, χ^2^(1) = 0.03, *p* = ns.

**Table 1:** Descriptive statistics for the two samples, test of gender differences, and bivariate correlations between the Shirom–Melamed Burnout Measure (SMB*M*) subscales and the overall SMBM index.

Sample 1: Adult workers (*N = 311*)							
	M	SD	Range	Skewness	Kurtosis	ANOVA	
Descriptive statistics						*F*	**η** ^2^
Physical exhaustion	2.72	1.26	1–7	0.87	0.35	0.05	0.000
Cognitive weariness	2.43	1.16	1–6	0.88	0.33	0.88	0.003
Emotional exhaustion	1.80	0.86	1–6	1.36	2.21	1.20	0.004
Overall SMB*M* Index	2.42	1.00	1–6.21	1.00	0.77	0.22	0.001
Bivariate correlations	1.	2.	3.	4.			
1. Physical exhaustion	–	0.75***	0.54***	0.93***			
2. Cognitive weariness	0.69***	–	0.68***	0.93***			
3. Emotional exhaustion	0.47***	0.55***	–	0.74***			
4. Overall SMB*M* Index	0.92***	0.89***	0.68***	–			
**Sample 2: Police officers (** ***N*** ** = 201)**							
	**M**	**SD**	**Range**	**Skewness**	**Kurtosis**	**ANOVA**	
Descriptive statistics						***F***	**η** **^2^**
Physical exhaustion	2.84	1.25	1–7	0.80	0.45	7.31**	0.035
Cognitive weariness	2.48	1.17	1–6.60	0.84	0.31	2.38	0.012
Emotional exhaustion	1.90	0.95	1–5.67	1.20	1.33	0.11	0.001
Overall SMB*M* Index	2.51	0.99	1–6	0.86	0.70	4.11*	0.020
Bivariate correlations	1.	2.	3.	4.			
1. Physical exhaustion	–	0.67***	0.51***	0.92***			
2. Cognitive weariness	0.53***	–	0.57***	0.89***			
3. Emotional exhaustion	0.52***	0.54***	–	0.71***			
4. Overall SMB*M* Index	0.88***	0.84***	0.73***	–			

Correlations for male participants are listed above the diagonal, correlations for female participants below the diagonal.

*p < 0.05, **p < 0.01, ***p< 0.001.

For the three SMB*M* subscales, the inter-item correlations were all above 0.20. Moreover, item-total correlations were all above the critical threshold of 0.40. In our sample, all Cronbach’s alpha values were satisfactory (physical exhaustion = 0.92, cognitive weariness = 0.95, emotional exhaustion = 0.90, SMB*M* overall index = 0.95).

Regarding convergent validity (**Table 2**), we found a positive correlation between the SMB*M* overall index and the PSS sum score (*r* = 0.56, *p* < 0.001). If compared to the emotional exhaustion subscale (*r* = 0.35, *p* < 0.001), we found stronger associations between the PSS sum score and the physical exhaustion (*r* = 0.54, *p* < 0.001) and cognitive weariness subscales (*r* = 0.49, *p* < 0.001). The SMB*M* indices were also moderately and positively correlated with the ERI ratio (*r* = 0.35–0.44, *p* < 0.001), the JDC ratio (*r* = 0.21–0.39, *p* < 0.001), and the depression subscale of the HADS (*r* = 0.38–0.53, *p* < 0.001).

**Table 2:** Bivariate correlations between burnout symptoms, perceived stress, depressive symptoms, and overall mental distress.

Sample 1: Adult workers (*N* = 311)				
	Physical exhaustion	Cognitive weariness	Emotional exhaustion	Overall SMB*M* Index
Perceived stress (PSS)	0.54*** (0.54***/0.55***)	0.49*** (0.47***/0.52***)	0.35*** (0.39***/0.33***)	0.56*** (0.56***/0.56***)
Effort–Reward Imbalance (ERI)	0.42*** (0.41***/0.44***)	0.37*** (0.33***/0.40***)	0.35*** (0.27**/0.42***)	0.44*** (0.41***/0.47***)
Job Demand-Control Imbalance (JDC)	0.39*** (0.30***/0.47***)	0.34*** (0.27**/0.42***)	0.21*** (0.12/0.32***)	0.39*** (0.30***/0.48***)
Depressive symptoms (HADS-D)	0.50*** (0.48***/0.53***)	0.47*** (0.47***/0.47***)	0.38*** (0.32***/0.43***)	0.53*** (0.51***/0.55***)
**Sample 2: Police officers (** ***N*** ** = 201)**				
	**Physical exhaustion**	**Cognitive weariness**	**Emotional exhaustion**	**Overall SMB** ***M*** ** Index**
Perceived stress (PSS)	0.59*** (0.74***/0.47***)	0.44*** (0.53***/0.37***)	0.42*** (0.42***/0.44***)	0.59*** (0.71***/0.49***)
Effort–Reward Imbalance (ERI)	0.33*** (0.31***/0.41***)	0.24*** (0.33**/0.20*)	0.14^+^ (0.11/0.15^+^)	0.31*** (0.34**/0.33***)
Job Demand-Control Imbalance (JDC)	0.28*** (0.37**/0.23**)	0.32*** (0.34**/0.31***)	0.12^+^ (0.24*/0.04***)	0.31*** (0.40**/0.26***)
Overall mental distress (GHQ-12)	0.56*** (0.49***/0.59***)	0.55*** (0.57***/0.53***)	0.43*** (0.29*/0.54***)	0.62*** (0.57***/0.64***)

SMBM, Shirom–Melamed Burnout Measure; PSS, Perceived Stress Scale; ERI, Effort Reward Imbalance; JDC, Job Demands and Control; HADS-D, Hospital Anxiety and Depression Scale–Depression Subscale; GHQ12, 12-item General Health Questionnaire. Correlations for female (first value) and male participants (second value) are listed in brackets.

+p < 0.10. *p < 0.05. **p < 0.01. ***p < 0.001.

**Figure 1:**
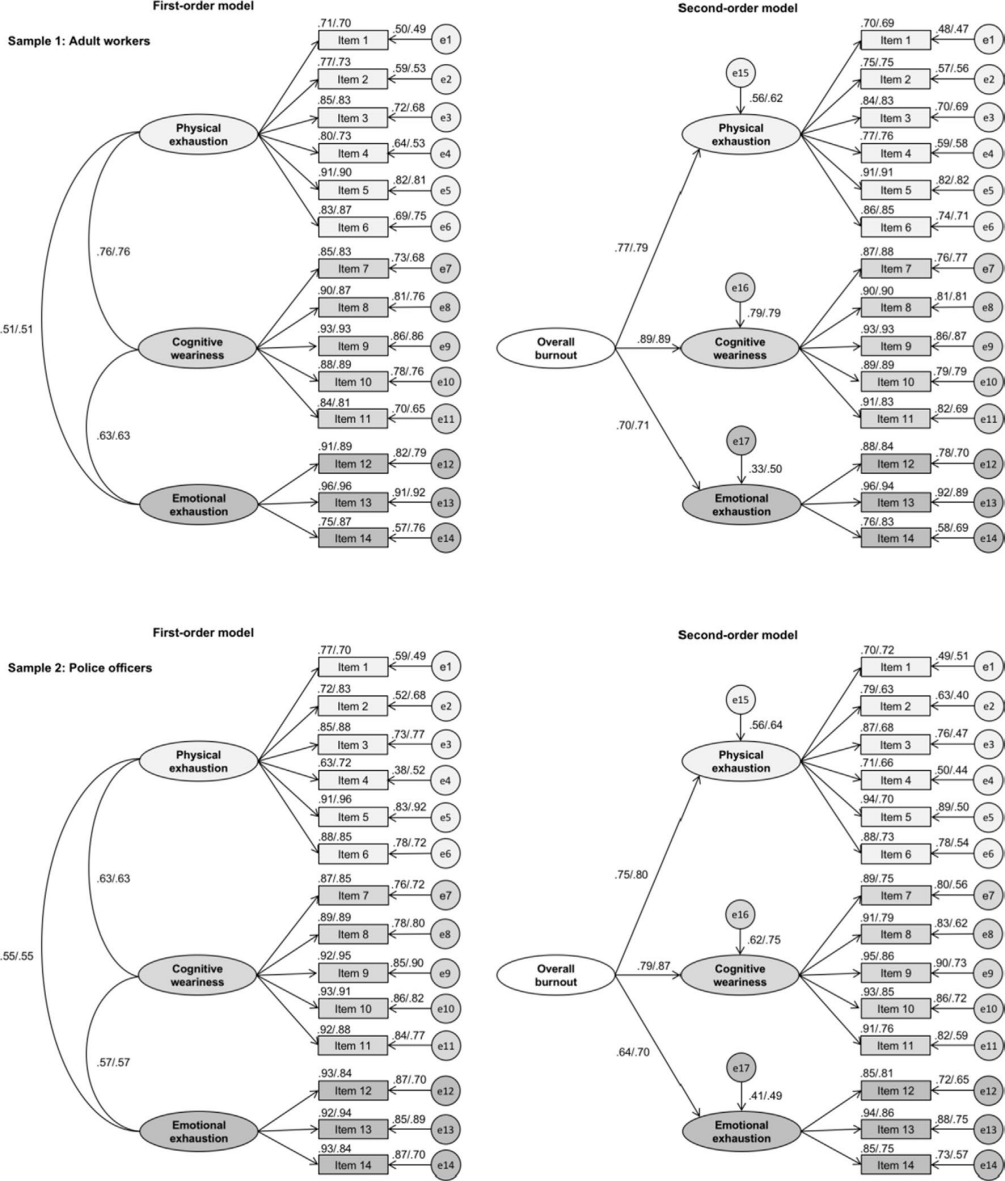
Factor loadings for confirmatory factor analysis for female (first coefficient) and male participants (second coefficient), for first-and second-order models, separately for adult workers and police officers.

Regarding factorial validity, we found a satisfactory model fit for the three-factor model for the first- and second-order model (**Table 3**). Configural and weak measurement invariance (invariant factor loadings) was supported across genders. The second-order model even supported strict measurement invariance. Factor loadings were very good (with all loadings being ≥0.63). The measurement coefficients for the three-factor models are displayed in **Figure 1**, both for female and male participants. We also found relatively strong associations between the SMB*M* subscales in the first-order model (*r* = 0.51–0.76, *p* < 0.001).

**Table 3:** Goodness-of-fit indices and model comparison.

	First-order model					Second-order model				
Sample 1: Adult workers (*N* = 311)	CFI	TLI	NFI	RMSEA	*p*(∆χ2)	CFI	TLI	NFI	RMSEA	*p*(∆χ2)
Default model	0.96	0.95	0.93	0.06 (0.05, 0.07)	–	0.97	0.96	0.94	0.06 (0.05, 0.07)	–
+ Configural invariance across genders	0.96	0.95	0.93	0.06 (0.05, 0.07)	0.105	0.97	0.96	0.94	0.05 (0.04, 0.06)	0.852
+ Weak invariance across genders	0.96	0.95	0.93	0.06 (0.05, 0.07)	0.226	0.97	0.96	0.94	0.05 (0.04, 0.06)	0.449
+ Strong invariance across genders	–	–	–	–	0.000	0.97	0.96	0.93	0.05 (0.04, 0.06)	0.485
+ Strict invariance across genders	–	–	–	–	–	0.97	0.97	0.93	0.05 (0.04, 0.06)	0.263
Sample 2: Police officers (*N* = 201)	CFI	TLI	NFI	RMSEA	*p*(∆χ2)	CFI	TLI	NFI	RMSEA	*p*(∆χ2)
Default model	0.97	0.97	0.93	0.05 (0.04, 0.07)	–	0.98	0.97	0.93	0.05 (0.04, 0.06)	–
+ Configural invariance across genders	0.97	0.97	0.93	0.05 (0.04, 0.06)	0.438	0.98	0.97	0.93	0.05 (0.03, 0.06)	0.853
+ Weak invariance across genders	0.97	0.97	0.92	0.05 (0.04, 0.06)	0.190	0.98	0.97	0.93	0.05 (0.03, 0.06)	0.391
+ Strong invariance across genders	–	–	–	–	0.000	–	–	–	–	0.022
+ Strict invariance across genders	–	–	–	–	–	–	–	–	–	–

AGFI, adjusted goodness-of-fit index; CFI, comparative fit index; TLI, Tucker Lewis index; RMR, root mean square residual; RMSEA, root mean square error of approximation; NFI, Normed Fit Index.

### Sample 2: Police Officers

#### Participants and Procedures

Sample 2 consisted of 201 police officers who were recruited from a police force in a bigger city in the Northwestern, German-speaking part of Switzerland. All officers (*N* = 980, 290 female, 690 male) were invited to participate in a comprehensive health check [including a cardiorespiratory fitness test, 7-day actigraphy, smartphone-based 2-day assessment of work-related affect and stressors, anthropometry, measurement of fasting blood lipid and blood glucose, blood pressure assessment, a computerized cognitive test (facial emotion recognition), a functional movement screen, a lung function test, and an online survey focusing on stress and mental health]. The health check was advertised *via* intranet, video clips on the internal TV channel, printed flyers, and verbal information during team meetings. Detailed information was given to all interested officers (e.g., about the voluntary basis of participation, no negative consequences in case of nonparticipation, information about benefits and risk, information about measurements). Out of all 980 information recipients, 201 participated in the study (20.5% response rate). Data were assessed between December 2017 and April 2018. A personalized health profile was given to the officers after the completion of the data assessment as an incentive for participation. Moreover, all officers had the opportunity to participate in a voluntary lifestyle coaching. All participants provided written informed consent before data assessment. All procedures were in line with the ethical principles described in the Helsinki Declaration, and approval was obtained for the study by the local ethics committee (EKNZ: Project-ID: 2017-01477).

#### Burnout

As with sample 1 (adult workers), we used the 14-item SMB*M* to measure burnout symptoms.

#### Perceived Stress

As with sample 1 (adult workers), police officers’ self-perceived levels of stress were assessed with the four-item PSS.

#### Occupational Stress

As for sample 1, occupational stress was assessed with the 11-item Job Content Questionnaire (JCQ) and the 16-item Effort-Reward Imbalance (ERI) questionnaire.

#### Overall Mental Distress

To assess overall mental distress, all officers filled in the German version of the General Health Questionnaire (GHQ-12) (70; 71). Participants were asked to rate their mental well-being, with reference to the previous week. Response options on a 4-point Likert scale ranged from 0 (not at all) to 3 (much more than usual). A sum score was calculated (from 0 to 36), with higher scores being reflective of higher levels of mental distress. Although no standard clinical cut-offs exist for the GHQ-12, researchers have used the following categories to successfully establish links between the GHQ-12 and mortality (if response options 0 + 1 = 0, and 2 + 3 = 1): asymptomatic (0), subclinically symptomatic (1–3), symptomatic (4–6), and highly symptomatic (7–12) (72, 73).

#### Statistical Analyses

We performed the same statistical analyses as with sample 1.

## Results

Sample 2 was composed of 72 female and 129 male participants. The mean age was *M* = 38.55 years (*SD* = 10.13; range, 22–62 years). Participants reported a mean job experience of *M* = 12.77 years (*SD* = 8.8; range, 0–37 years). All participants were employed for at least 30% (*M* = 92.08%; *SD* = 18.21; range, 30–100%), with 79.6% having a full-time employment. The sample had a mean body mass index (height in cm/body weight in kg^2^) of *M* = 25.77 (*SD* = 3.63), with 52.7% of the sample (*n* = 106) being classified as overweight (BMI ≥ 25). Moreover, 43.3% (*n* = 87) reported that they have children living at home, 3.0% (*n* = 6) had responsibility as a caregiver for a person in need of care, and 48.3% (*n* = 97) reported shift work. With regard to participants’ highest level of education, 2.0% (*n* = 4) finished compulsory school without additional training, 50.7% (*n* = 102) completed vocational education and training, 7.5% (*n* = 15) completed an academic high school, and 39.8% (*n* = 80) completed higher education. Finally, 18.4% (*n* = 37) reported that they are smokers, whereas 10.0% (*n* = 20) reported taking antidepressant medication.

In the total sample, we found a score for the SMB*M* overall index of 2.51 (*SD* = 0.99) (**Table 1**). Moreover, 5% (*n* = 9) of the participants had burnout levels above the cut-off for clinically relevant burnout symptoms (≥4.40). In comparison to male participants, female officers scored higher with regard to physical exhaustion (women: *M* = 3.15, *SD* = 1.30; men: *M* = 2.66, *SD* = 1.19) and the overall SMB*M* index (women: *M* = 2.70, *SD* = 1.00; men: *M* = 2.40, *SD* = 0.96). The distribution of men (*n* = 6, 4.7%) and women (*n* = 3, 4.2%) was similarly among those participants above the cut-off for clinically relevant burnout symptoms, χ^2^(1) = 0.03, *p* = ns.

The inter-item correlations exceeded the critical value of 0.20, and all item-total correlations were above the threshold of 0.40, for each of the three SMB*M* subscales. The Cronbach’s alpha values were satisfactory across all SMBM indices (physical exhaustion = 0.92, cognitive weariness = 0.95, emotional exhaustion = 0.90, SMBM overall index = 0.95).

With respect to convergent validity (**Table 2**), we observed positive associations between the SMB*M* (subscales and overall index), self-perceived stress (*r* = 0.42–0.59), the ERI ratio (*r* = 0.14–0.31), and the JDC ratio (*r* = 0.12–0.32). Only a statistically nonsignificant trend towards a positive relationship was found between emotional exhaustion and the ERI/JDC ratios. Finally, overall mental distress was positively associated with all SMBM indices (*r* = 0.43–0.62, *p* < 0.001).

A good model fit was found for the three-factor CFA model. Moreover, both the first- and second-order model were supported (**Table 3**). Evidence for weak measurement invariance (invariant factor loadings) across genders was supported. As shown in **Figure 1**, very good factor loadings were observed across all items (all loadings ≥ 0.63), both for women and men. With regard to the first-order model, the three SMB*M* subscales were strongly correlated with each other (*r* = 0.55–0.63, *p* < 0.001).

## Discussion

The present studies show that the German version of the SMB*M* has adequate psychometric properties and acceptable convergent validity and can therefore be used in burnout research in various samples of adult workers. Moreover, the factor structure of the SMB*M* was supported in CFA and found to be gender invariant. This work expands the current literature in an important way in that we, for the first time, thoroughly examined the validity of the SMBM among adult workers and examined whether the instrument performs equally well in male and female participants. Given that the SMBM is among the most widely used instruments to assess burnout symptoms, such an analysis seemed highly warranted. Based on the study aims, four hypotheses were formulated; below, each hypothesis is discussed in detail.

With the first hypothesis we expected that, compared to male participants, female participants would report higher burnout symptoms, and data from police officers confirmed this. However, contrary to our hypothesis, no gender differences were found in the broader sample of adult workers, which is at odds with prior research in adult populations (50, 57). How to explain this unexpected pattern of results? While in our adult sample, no gender difference existed with regard to age, BMI, marital status, children at home, caregiving, job experience, educational level, smoking status, and use of medication, we found that men had a higher mean employment rate than women (96.2% vs. 78.84% in full-time employment). Therefore, it can be speculated that the lack of gender differences might be attributed to lower employment rates among women. However, a higher full-time employment rate was also found among male (90%) compared to female (56%) participants in our sample of police officers. An alternative explanation might be that burnout levels were generally low in the sample of adult workers, with only 5.8% reporting clinically relevant burnout symptoms (46). However, we acknowledge that generally low burnout levels were also observed in our sample of police officers. With regard to the low burnout levels, we argue that the recruitment strategies to address adult workers and police officers might have led to a selection bias in the sense that students more often contacted healthy people, and/or that healthy people were more willing to participate in the study. Finally, in line with previous studies (24, 57), higher subscale mean scores were found for physical fatigue in both samples and genders if compared to cognitive weariness and emotional exhaustion.

With the second hypothesis, we expected that internal consistency of the SMB*M* would be satisfactory in both adult workers and police officers and both women and men (23, 24, 32), and generally, our data confirmed this. Thus, all Cronbach’s alpha coefficients exceeded ≥0.70, for all SMB*M* indices, in both male and female participants, and across both samples. Moreover, we found inter-item correlations of ≥0.20 within the respective factor for both male and female participants. All item-total correlations exceeded the critical value of 0.40. Finally, according to the standards recommended by West et al. (74), we observed that the descriptive statistics met the prerequisites for parametric tests, with skewness being <2 and kurtosis being <7 across all SMB*M* indices.

With the third hypothesis we expected to find evidence for the convergent validity of the SMB*M* in both male and female participants. Full support was found for this hypothesis. In accordance with previous studies (52, 50, 55), the SMB*M* overall index was at least moderately and positively associated with participants’ levels of perceived stress. In our two populations, we also found weak-to-moderate (positive) correlations between the SMB*M* indices and occupational stress, which accords well with previous research in this area (75–77). The observation that stronger correlations were found for the PSS can be explained by the fact that the PSS is a general measure of stress, whereas the ERI and JDC ratios assess specific forms of occupational stress that might not be applicable for some participants. Moreover, our results corroborate prior research, in which at least moderate correlations were observed between the SMB*M* overall index and mental health outcomes such as depressive symptoms (44, 45, 48). The correlations were moderate-to-strong in both populations, with slightly higher correlations found in police officers. However, these differences are difficult to interpret because we used different instruments to assess mental health in each sample. In the adult worker sample, our findings suggest that the SMB*M* overall index and depressive symptoms have 26.0% (women) and 30.3% (men) of common variance. As highlighted by Melamed et al. (27), it can be expected that burnout and depressive symptoms have a certain overlap because they share some characteristic features such as fatigue and loss of energy.

Support was also found for our fourth and final hypothesis, that the three-factorial model would fit well with the empirical data: The findings of our studies indicate that a three-factor model provided an excellent model fit across all samples, with all factor loadings being strongly linked to the respective factors (24). The first-order model showed that the latent factors were moderately to highly correlated with each other (*r* = 0.51–0.76, *p* < 0.001) [cp. Refs, (19, 23, 57)]. Following Shirom and Melamed (19), this magnitude of correlations between the SMB*M* subscales is to be expected because every individual possesses a pool of energetic resources. These resources are closely interrelated, and a deficit in one resource can lead to deficits in other resources. Moreover, in line with a previous study with Canadian workers (24), our findings support the factorial validity of a second-order model. This lends further support to the notion that it is legitimate to use the SMB*M* overall score as a global/general burnout index. Sassi and Neveu (24) found that overall burnout explained 63, 53, and 27% of variance in physical exhaustion, cognitive weariness, and emotional exhaustion, respectively, which was comparable to the findings reported in our analyses (physical exhaustion, 56–64%; cognitive weariness, 62–79%; emotional exhaustion, 33–50%). The fact that an adequate model fit was found for the second-order model indicates that the items of the SMB*M* subscales can be aggregated to form an overall burnout index. Finally, for the first time, our results provide evidence for weak-to-strict measurement invariance across male and female workers. This is an important finding because Widaman et al. (69) argued that, if participants’ answers vary so much as a function of gender that significant differences emerge in the factor structure of that instrument, the measuring device must change. The same would be the case for relevant ceiling or floor effects occurring for male and female participants. Thus, the present analyses suggest that the SMB*M* is equally suitable to assess burnout symptoms independent of participants’ gender.

Despite the novelty of our study, some methodological shortcomings should be mentioned that might limit the generalizability of our data: First, the cross-sectional nature of our studies did not allow us to examine test–retest reliability and predictive validity. Second, the correlations reported in the present samples were not controlled for other demographic factors, although previous studies have shown that participants with elevated burnout are more often divorced, blue-collar workers, have lower education levels, are foreigners, unemployed, financially strained, use more medication, and report less healthy behaviors (50, 52, 54). Third, both samples consisted of nonclinical populations. Therefore, it was not possible to examine the discriminant validity of the SMB*M*, particularly as the number of participants with clinically relevant burnout levels was low in the present study. Accordingly, we were unable to test the discriminant validity of the cut-off for clinically relevant burnout (≥4.40), which Lundgren-Nilsson et al. (20) previously suggested for the SMBQ. This is an important shortcoming that should be addressed in future research. Fourth, while for study 1, we attempted to recruit a sample of adult workers that is broad in terms of employment, education, age, and gender, we did not assess specific information about the participants’ occupations. Thus, we were not able to examine whether the described relationships differ according to varying professions. Finally, we acknowledge that there are other validated instruments to assess burnout symptoms in German-speaking (and international) populations. One such instrument is the Oldenburg Burnout Inventory (OLBI) (78–80), in which burnout symptoms are operationalized via two dimensions (exhaustion, disengagement from work). As it applies for the SMBM (14 items, based on COR theory), it is a particular strength of the OLBI that the instrument is concise (16 items) and that it has been developed based on a solid theoretical foundation (Job Demands–Resources model of burnout). We therefore suggest that this instrument could be used in future research to test the discriminant validity of the SMB*M*. Specifically, we would expect that the SMB*M* scales are more strongly correlated with the exhaustion than the disengagement from work subscale of the OLBI. A strong correlation can be expected with the exhaustion subscale because, as in the SMB*M*, the OLBI assesses affective, cognitive, and physical aspects of exhaustion.

## Conclusions and Practical Relevance

The SMB*M* is among the most widely used tools in international burnout research. Our study shows, for the first time, that the German version of the instrument has adequate psychometric properties and satisfactory convergent and factorial validity in a broad sample of adult workers and police officers. The SMB*M* can provide relevant information for screening and treatment planning. More research is needed to establish the validity of the cut-off score for clinically relevant burnout. This is essential for finding out whether the SMB*M* can be used in the early screening process to identify employees who might suffer from clinically significant burnout symptoms.

## Data Availability

The datasets used and/or analyzed during the current study are available from the corresponding author on reasonable request *via* the Ethics Committee of Northwestern and Central Switzerland (EKNZ), Ms. Nienke Jones (Nienke.jones@bs.ch; +41 61 268 13 54). At the time of obtaining ethical clearance for the present study from the EKNZ, and in line with Swiss laws, we stated that only authorized researchers who are directly involved in the present project will have access to the raw data. Accordingly, and in line with this statement, we cannot grant access to the data for third parties, unless this is officially approved by the EKNZ.

## Ethics Statement

All procedures were in line with the ethical principles described in the Helsinki Declaration. The data collection and treatment of the participants is in line with the APA ethical standards. The Ethics Committee for Northwest/Central Switzerland (Ethikkommission Nordwest- und Zentralschweiz) approved the studies of both samples. Sample 1: Written informed consent was obtained from all participants prior to data assessment (study approval number: EKNZ: 240/12). Sample 2: All participants provided written informed consent prior to data assessment (study approval number: EKNZ: Project-ID: 2017-01477).

## Author Contributions

RS, SB, and MG made substantial contributions to conception and design of the study. SL and RS were responsible for the acquisition of data. RS, FC, and MG were responsible for the analysis and interpretation of data. RS and MG drafted the manuscript. FC and SB wrote sections of the manuscript. SB, SL, and FC critically reviewed and revised the initial draft. All authors have approved the final version of the submitted manuscript.

## Conflict of Interest Statement

The authors declare that the research was conducted in the absence of any commercial or financial relationships that could be construed as a potential conflict of interest.

## References

[1] Shirom A Job-related burnout. In Quick J. C. Tetrick L. E. (Eds). In: Handbook of occupational health psychology. Washington: American Psychological Association (2003). p. 245–65. 10.1037/10474-012

[2] Bianchi R Boffy C Hingray C Truchot D Laurent E Comparative symptomatology of burnout and depression. J Health Psychol (2013) 18:782–7. 10.1177/1359105313481079 23520355

[3] Bianchi R Verkuilen J Brisson R Schonfeld IS Laurent E Burnout and depression: label-related stigma, help-seeking, and syndrome overlap. Psychiatry Res (2016) 245:91–8. 10.1016/j.psychres.2016.08.025 27529667

[4] Laurent E Bianchi R Schonfeld IS Vandel P Editorial: depression, burnout, and other mood disorders: interdisciplinary approaches. Front Psychol (2017) 8:282. 10.3389/fpsyg.2017.00282 28321197 PMC5337502

[5] Bianchi R Schonfeld IS Laurent E Burnout or depression: both individual and social issue. Lancet (2017a) 390:230. 10.1016/S0140-6736(17)31606-9 28721879

[6] Bianchi R Schonfeld IS Laurent E Can we trust burnout research? Ann Oncol (2017b) 28:2320–1. 10.1093/annonc/mdx267 28520921

[7] Bianchi R Schonfeld IS Laurent E On the overlap of vital exhaustion and depression. Eur Psychiatry (2017c) 44:161–3. 10.1016/j.eurpsy.2017.04.007 28641218

[8] Bianchi R Schonfeld IS Laurent E Physician burnout is better conceptualized as depression. Lancet (2017d) 389:1397–8. 10.1016/S0140-6736(17)30897-8 28402821

[9] Maslach C Leiter MP Understanding the burnout experience: recent research and its implications for psychiatry. World Psychiatry (2016) 15:103–11. 10.1002/wps.20311 PMC4911781 27265691

[10] Chirico F Burnout syndrome and depression are not the same thing. Br J Psychiatry (2017a) 190:1–2. 10.1192/bjp.1190.1191.1181a

[11] Chirico F Is it time to consider burnout syndrome an occupational disease? Br J Psychiatry (2017c) 190:1. 10.1192/bjp.1190.1191.1181a

[12] Weber A Jaekel-Reinhard A Burnout syndrome: a disease of modern societies? Occup Med (Lond) (2000) 50:512–7. 10.1093/occmed/50.7.512 11198677

[13] American Psychological Association (2013). The diagnostic and statistical manual of mental disorders (DSM-5): PTSD fact sheet. Washington, D.C: American Psychiatric Association. 10.1176/appi.books.9780890425596

[14] Glise K Hadzibajramovic E Jonsdottir IH Ahlborg G Jr. Self-reported exhaustion: a possible indicator of reduced work ability and increased risk of sickness absence among human service workers. Int Arch Occup Environ Health (2010) 83:511–20. 10.1007/s00420-009-0490-x 19943058

[15] Glise K Ahlborg GJ Jonsdottir IH Course of mental symptoms in patients with stress-related exhaustion: does sex or age make a difference. BMC Psychiatry (2012) 12:1–18. 10.1186/1471-244X-12-18 22409935 PMC3338076

[16] Lastovkova A Burnout syndrome as an occupational disease in the European Union: an exploratory study. Ind Health (2018) 56:160–5. 10.2486/indhealth.2017-0132 PMC5889935 29109358

[17] Chirico F Is burnout a syndrome or an occupational disease?Instructions for occupational physicians. Epidemiol Prev (2017b) 41:294–8. 10.19191/EP17.5-6.P294.089 29119764

[18] Shirom A Melamed S Toker S Berliner S Shapira I Burnout and health review: current knowledge and future research directions. Int Rev Ind Organ Psychol (2006) 20:269–309. 10.1002/0470029307.ch7

[19] Shirom A Melamed S A comparison of the construct validity of two burnout measures in two groups of professionals. Int J Stress Manag (2006) 13:176–200. 10.1037/1072-5245.13.2.176

[20] Lundgren-Nilsson A Jonsdottir IH Pallant J Ahlborg G Internal construct validity of the Shirom–Melamed Burnout Questionnaire (SMBQ). BMC Public Health (2012) 12(1):1. 10.1186/1471-2458-12-1 22214479 PMC3307433

[21] Maslach C Schaufeli WB Leiter MP Job burnout. Annu Rev Psychol (2001) 52:397–422. 10.1146/annurev.psych.52.1.397 11148311

[22] Maslach C Leiter MP The truth about burnout. San Francisco: Jossey-Bass (1997).

[23] Qiao H Schaufeli WB The convergent validity of four burnout measures in a Chinese sample: a confirmatory factor-analytic approach. Appl Psychol (2011) 60:87–111. 10.1111/j.1464-0597.2010.00428.x

[24] Sassi N Neveu J-P Traduction et validation d’une nouvelle mesure d’épuisement professionnel: le Shirom–Melamed Burnout Measure. Can J Behav Sci (2010) 42:177–84. 10.1037/a0017700

[25] Hobfoll SE Shirom A Stress and burnout in the workplace: conservation of resources. In: Golembiewski RT, editor. Handbook of organizational behavior. Dekker (1993). p. 41–61.

[26] Hobfoll SE Shirom A Conservation of resources theory: applications to stress and management in the workplace. In: Golembiewski RT, editor. Handbook of organization behavior. Dekker (2000). p. 57–81.

[27] Melamed S Shirom A Toker S Berliner S Shapira I Burnout and risk of cardiovascular disease: evidence, possible causal paths, and promising research directions. Psychol Bull (2006) 132:327–53. 10.1037/0033-2909.132.3.327 16719565

[28] Hobfoll SE Conservation of resources: a new attempt at conceptualizing stress. Am Psychol (1989) 44:513–24. 10.1037//0003-066X.44.3.513 2648906

[29] Hobfoll SE Stress, culture, and community. In: The psychology and philosophy of stress. New York: Plenum Press (1998).

[30] Kushnir T Melamed S The Gulf War and its impact on burnout and well-being of working civilians. Psychol Med (1992) 22:987–95. 10.1017/S0033291700038551 1488493

[31] Melamed S Kushnir T Shirom A Burnout and risk factors for cardiovascular disease. Behav Med (1992) 18:53–60. 10.1080/08964289.1992.9935172 1392214

[32] Lerman Y Melamed S Shragin Y Kushnir T Rotgoltz Y Shirom A Association between burnout at work and leukocyte adhesiveness/aggregation. Psychosom Med (1999) 61:828–33. 10.1097/00006842-199911000-00017 10593635

[33] Melamed S Ugarten U Shirom A Kahana L Lerman Y Froom P Chronic burnout, somatic arousal and elevated cortisol levels. J Psychosom Res (1999) 46:591–8. 10.1016/S0022-3999(99)00007-0 10454175

[34] Shirom A Westman M Shamai O Carel RS Effects of work overload and burnout on cholesterol and triglycerides levels: the moderating effects of emotional reactivity among male and female employees. J Occup Health Psychol (1997) 2:275–88. 10.1037//1076-8998.2.4.275 9552297

[35] Grossi G Perski A Evengard B Blomkvist V Orth-Gomer K Physiological correlates of burnout among women. J Psychosom Res (2003) 55:309–16. 10.1016/S0022-3999(02)00633-5 14507541

[36] Gerber M Börjesson M Ljung T Lindwall M Jonsdottir I Fitness moderates the relationship between stress and cardiovascular risk factors. Med Sci Sports Exerc (2016) 48:2075–81. 10.1249/MSS.0000000000001005 27285493

[37] Grossi G Perski A Akstedt M Johannson T Lindström M Holm K The morning salivary cortisol response in burnout. J Psychosom Res (2005) 59:103–11. 10.1016/j.jpsychores.2005.02.009 16186006

[38] Toker S Shirom A Shapira I Berliner S Melamed S The association between burnout, depression, anxiety, and inflammation biomarkers: C-reactive protein and birinogen in men and women. J Occup Health Psychol (2005) 10:344–62. 10.1037/1076-8998.10.4.344 16248685

[39] Melamed S Shirom A Froom P Burnout and risk of type 2 diabetes mellitus (DM) in Israeli workers. In Work, Stress and Health Conference. Toronto, ON, Canada (2003) 3: p. 20–22.

[40] Melamed S Shirom A Toker S Shapira I Burnout and risk of type 2 diabetes: a prospective study of apparently healthy employed persons. Psychosom Med (2006) 68:863–9. 10.1097/01.psy.0000242860.24009.f0 17132837

[41] Soares JJ Jablonska B Psychological experiences among primary care patients with and without musculoskeletal pain. Eur J Pain (2004) 8:79–89. 10.1016/S1090-3801(03)00083-1 14690678

[42] Sheiner E Sheiner E Carel R Potashnik G Shoham-Vardi I Potential association between male infertility and occupational psychological stress. J Occup Environ Med (2002) 44:1–7. 10.1097/00043764-200212000-00001 12500450

[43] Nordin M Akerstedt T Nordin S Psychometric evaluation and normative data for the Karolinska Sleep Questionnaire. Sleep Biol Rhythm (2013) 11:216–26. 10.1111/sbr.12024

[44] Gerber M Lang C Feldmeth AK Elliot C Brand S Holsboer-Trachsler E Pühse U Burnout and mental health in Swiss vocational students: the moderating role of physical activity. J Res Adolesc (2015) 25(1): 63–74. 10.1111/jora.12097

[45] Toker S Biron M Job burnout and depression: unraveling their temporal relationship and considering the role of physical activity. J Appl Psychol (2012) 97:699–710. 10.1037/a0026914 22229693

[46] Gerber M Jonsdottir IH Lindwall M Ahlborg G Physical activity in employees with differing occupational stress and mental health profiles: a latent profile analysis. Psychol Sport Exerc (2014) 15:649–58. 10.1016/j.psychsport.2014.07.012

[47] Gerber M Lang C Feldmeth AK Elliot C Brand S Hoslboer-Trachsler E Burnout and mental health in Swiss vocational students: the moderating role of physical activity. J Res Adolesc (2015) 25:63–74. 10.1111/jora.12097

[48] Schonfeld IS Bianchi R Burnout and depression: two entities or one? J Clin Psychol (2016) 72:22–37. 10.1002/jclp.22229 26451877

[49] Lindegård A Jonsdottir IH Börjesson M Lindwall M Gerber M Changes in mental health in compliers and non-compliers with physical activity recommendations in patients with stress-related exhaustion. BMC Psychiatry (2015) 15:1–10. 10.1186/s12888-015-0642-3 26530329 PMC4632342

[50] Norlund S Reuterwall C Höög J Lindahl B Janlert U Slunga Birgander L Burnout, working conditions and gender—results from the northern Sweden MONICA Study. BMC Public Health (2010) 10:1–9. 10.1186/1471-2458-10-326 20534136 PMC2896942

[51] Jonsdottir IH Rödjer L Hadzibajramovic E Börjesson M Ahlborg GJ A prospective study of leisure-time physical activity and mental health in Swedish health care workers and social insurance officers. Prev Med (2010) 51:373–7. 10.1016/j.ypmed.2010.07.019 20691721

[52] Soares JJ Grossi G Sundin O Burnout among women: associations with demographic/socio-economic, work, life-style and health factors. Arch Womens Ment Health (2007) 10:61–71. 10.1007/s00737-007-0170-3 17357826

[53] Nunnally J Bernstein B Psychometric theory. New York: McGraw-Hill (1994).

[54] Lindwall M Gerber M Jonsdottir I Börjesson M Ahlborg GJ The relationships of change in physical activity with change in depression, anxiety, and burnout: a longitudinal study of Swedish healthcare workers. Health Psychol (2014) 33:1309–18. 10.1037/a0034402 24245832

[55] Gerber M Lindwall M Lindegård A Börjesson M Jonsdottir IH Cardiovascular fitness protects from stress-related symptoms of burnout and depression. Patient Educ Couns (2013) 93:146–52. 10.1016/j.pec.2013.03.021 23623176

[56] Swami MK Mathur DM Pushp BK Emotional intelligence, perceived stress and burnout among resident doctors: an assessment of the relationship. Natl M J India (2013) 26:210–3. 24758443

[57] Böhm D Stock Gissendanner S Finkeldey F John SM Werfel T Diepgen TL Severe occupational hand eczema, job stress and cumulative sickness absence. Occup Med (2014) 64:509–15. 10.1093/occmed/kqu076 24994848

[58] Elliot C Lang C Brand S Holsboer-Trachsler E Pühse U Gerber M The relationship between meeting vigorous physical activity recommendations and burnout symptoms among adolescents: an exploratory study with vocational students. J Sport Exerc Psychol (2015) 37:180–92. 10.1123/jsep.2014-0199 25996108

[59] Gerber M Feldmeth AK Lang C Brand S Elliot C Holsboer-Trachsler E The relationship between mental toughness, stress, and burnout among adolescents: a longitudinal study with Swiss vocational students. Psychol Rep (2015) 117:703–23. 10.2466/14.02.PR0.117c29z6 26652888

[60] Comrey AL Lee HB A first course in factor analysis. Hillsdale: Erlbaum (1992).

[61] Cohen S Kamarck T Mermelstein R A global measure of perceived stress. J Health Soc Behav (1983) 24:385–96. 10.2307/2136404 6668417

[62] Leung DYP Lam TH Chan SSC Three versions of perceived stress scale: validation in a sample of Chinese cardiac patients who smoke. BMC Public Health (2010) 10:513–9. 10.1186/1471-2458-10-513 PMC2939644 20735860

[63] Gerber M Kalak N Lemola S Clough PJ Perry JL Pühse U Are adolescents with high mental toughness levels more resilient against stress? Stress Health (2013) 29:164–71. 10.1002/smi.2447 22941714

[64] Karasek R Brisson C Kawakami N Houtman I Bongers P Amick B The Job Content Questionnaire (JCQ): an instrument for internationally comparative assessments of psychosocial job characteristics. J Occup Health Psychol (1998) 3:322–55. 10.1037//1076-8998.3.4.322 9805280

[65] Siegrist J Wege N Pühlhofer F Wahrendorf M A short generic measure of work stress in the era of globalization: effort–reward imbalance. Int Arch Occup Environ Health (2009) 82:1005–13. 10.1007/s00420-008-0384-3 19018554

[66] Zigmond AS Snaith RP The hospital anxiety and depression scale. Acta Psychiatr Scand (1983) 67:361–70. 10.1111/j.1600-0447.1983.tb09716.x 6880820

[67] McDonald RP Ho RM Principles and practice in reporting structural equation analyses. Psychol Methods (2002) 7:65–9. 10.1037//1082-989X.7.1.64 11928891

[68] Byrne BM Structural equation modeling with AMOS. In: Basic concepts, applications, and programming. New York: Taylor & Francis (2010).

[69] Widaman KF Ferrer E Conger RD Factorial invariance within longitudinal structural equation models: measuring the same construct across time. Child Dev (2010) 4:10–8. 10.1111/j.1750-8606.2009.00110.x PMC2848495 20369028

[70] Schmitz N Kruse J Tress W Psychometric properties of the General Health Questionnaire (GHQ-12) in a German primary care sample. Acta Psychiatr Scand (1999) 100(6): p. 462–8. 10.1111/j.1600-0447.1999.tb10898.x 10626926

[71] Romppel M Braehler E Roth M Glaesmer H What is the General Health Questionnaire-12 assessing? Dimensionality and Psychometric Properties of the General Health Questionnaire-12 in a Large Scale German Population Sample. Compr Psychiatry, (2013) 54(4): p. 406–13. 10.1016/j.comppsych.2012.10.010 23206494

[72] Puustinen PJ Koponen H Kautiainen H Mäntyselkä P Vanhala M Psychological distress measured by the GHQ-12 and mortality: a prospective population-based study. Scand J Public Health (2011) 39:577–81. 10.1177/1403494811414244 21752849

[73] Russ TC Stamatakis E Hamer M Starr JM Kivimäki M Batty GD Association between psychological distress and mortality: individual participant pooled analysis of 10 prospective cohort studies. BMJ (2012) 345. 10.1136/bmj.e4993 PMC3409083 22849956

[74] West SG Finch JF Curran PJ Structural equation models with nonnormal variables: problems and remedies. In: Hoyle RH, editor. Structural equation modeling. Concepts, issues, and applications. Thousand Oaks: Sage (1995). p. 56–75.

[75] Garbarino S Magnavita N Elovainio M Heponiemi T Ciprani F Cuomo G Police job strain during routine activities and a major event. Occup Med (2011) 61:395–9. 10.1093/occmed/kqr058 21642475

[76] Garbarino S Cuomo G Chiorri C Magnavita N Association of work-related stress with mental health problems in a special police force unit. BMJ Open (2013) 19. 10.1136/bmjopen-2013-002791 PMC3717472 23872288

[77] Chirico F Job stress models for predicting burnout syndrome: a review. Ann Ist Super Sanità (2016) 52:443–56. 10.4415/ANN_16_03_17 27698304

[78] Demerouti E Bakker AB Nachreiner F Schaufeli WB The Job Demands–Resources model of burnout. J Appl Psychol (2001) 86:499–512. 10.1037//0021-9010.86.3.499 11419809

[79] Demerouti E Bakker AB Vardakou I Kantas A The convergent validity of two burnout instruments: a multitrait–multimethod analysis. Eur J Psychol Assess (2003) 18:296–307.

[80] Halbesleben JRB Demerouti E The construct validity of an alternative measure of burnout: investigating the English translation of the Oldenburg Burnout Inventory. Work Stress (2005) 19:208–20. 10.1080/0267837050034072

