# Supplementary material for: Psychometric Properties and Convergent Validity of the Shirom–Melamed Burnout Measure in Two German-Speaking Samples of Adult Workers and Police Officers
Source: Front Psychiatry. 2019 Aug 2;10:536. doi: 10.3389/fpsyt.2019.00536 (PMC6688652; doi:10.3389/fpsyt.2019.00536)
Supplement: Supplementary file 1 [file DataSheet_1.pdf]

Shirom-Melamed Burnout Measure (SMBM)

Wie fühlen Sie sich bei der Arbeit?

Im Folgenden finden Sie eine Reihe von Aussagen, die verschieden Gefühle beschreiben, die Sie bei der Arbeit erleben könnten. Bitte geben Sie an, wie oft Sie in den letzten 30 Arbeitstagen jedes der folgenden Gefühle gehabt haben:

Wie oft haben Sie sich bei der Arbeit so gefühlt?

		Nie oder fast nie	Sehr selten	Ziemlich selten	Manchmal	Ziemlich oft	Sehr oft	Immer oder fast immer
P	1. Ich fühle mich müde.	1	2	3	4	5	6	7
P	2. Ich habe keine Energie, um morgens zur Arbeit zu gehen.	1	2	3	4	5	6	7
P	3. Ich fühle mich körperlich ausgelaugt.	1	2	3	4	5	6	7
P	4. Ich habe die Nase voll.	1	2	3	4	5	6	7
P	5. Ich habe das Gefühl, dass meine Batterien leer sind.	1	2	3	4	5	6	7
P	6. Ich fühle mich ausgebrannt.	1	2	3	4	5	6	7
K	7. Mein Denken ist verlangsamt.	1	2	3	4	5	6	7
K	8. Ich habe Schwierigkeiten, mich zu konzentrieren.	1	2	3	4	5	6	7
K	9. Ich habe das Gefühl, nicht klar zu denken.	1	2	3	4	5	6	7
K	10. Ich habe das Gefühl, beim Denken nicht bei der Sache zu sein.	1	2	3	4	5	6	7
K	11. Ich habe Schwierigkeiten, über komplexe Dinge nachzudenken.	1	2	3	4	5	6	7
E	12. Ich fühle mich nicht in der Lage, mich auf die Bedürfnisse von Arbeitskollegen und Kunden einzustellen.	1	2	3	4	5	6	7
E	13. Ich fühle mich nicht in der Lage, gefühlsmässig in Arbeitskollegen oder Kunden zu investieren.	1	2	3	4	5	6	7
E	14. Ich fühle mich nicht in der Lage, mich in Arbeitskollegen oder Kunden hineinzuversetzen.	1	2	3	4	5	6	7

Beachte:

Die Buchstaben vor jeder Frage stehen für die 3 Subskalen des Shirom-Melamed-Burnout Measure (SMBM).

P = physische Mattigkeit

K = kognitive Ermüdung

E = emotionale Erschöpfung

German version 20 Oct 2007; B.Schulze, University of Zurich, Switzerland

Ver 2. july 2005
